# Dual CXCR4/IL-10 Gene-Edited Human Amniotic Mesenchymal Stem Cells Exhibit Robust Therapeutic Properties in Chronic Wound Healing

**DOI:** 10.3390/ijms232315338

**Published:** 2022-12-05

**Authors:** Seong-Ho Han, Dong-Sik Chae, Sung-Whan Kim

**Affiliations:** 1 Department of Family Medicine, Dong-A University College of Medicine, Dong-A University Medical Center, Busan 49201, Republic of Korea; 2 Department of Orthopedic Surgery, Catholic Kwandong University College of Medicine, International St. Mary’s Hospital, Incheon 22711, Republic of Korea; 3 Department of Medicine, Catholic Kwandong University College of Medicine, Gangneung 25601, Republic of Korea

**Keywords:** cell therapy, CXCR4, IL-10, gene editing, mesenchymal stem cells, wound healing

## Abstract

Although stem cells have attracted attention as a novel therapeutic solution for tissue regeneration, their minimal efficacy remains controversial. In the present study, we aimed to investigate the enhanced therapeutic property of *CXCR4/IL-10* dual angiogenic/anti-inflammatory gene knock-in amniotic mesenchymal stem cells (AMM) in a wound-healing model. Dual *CXCR4* and *IL-10* genes were inserted into the AMM genome using transcription-activator-like effector nuclease (TALEN). Matrigel tube formation and anti-inflammatory effects were assessed in vitro, and efficacy was tested in vivo in a diabetic wound-healing model. CXCR4/IL-10-expressing amniotic MSCs (AMM/CI) strongly expressed *CXCR4* and *IL-10* genes and robustly promoted tube formation and anti-inflammatory potential. AMM/CI transplantation resulted in accelerated wound healing, as well as high engraftment and re-epithelialization potential. Transplanted AMM/CI also exhibited high angiogenic and decreased pro-inflammatory gene expression in the wound tissue, indicating direct therapeutic effects on wound healing. Taken together, these data indicate that dual angiogenic/anti-inflammatory gene knock-in may be a novel approach to enhance the therapeutic effects of stem cells, and transplantation of AMM/CI can be an alternative therapeutic option in chronic wound healing.

## 1. Introduction

The skin plays a pivotal role in protecting the body against multiple pathogens. Many chronic diseases, including diabetes, burns, and peripheral arterial disease, can cause refractory wounds. Chronic wounds usually fail to heal within three months and show a continuous inflammatory state [1]. The incidence of chronic wounds is rapidly increasing in Westernized countries owing to obesity and diabetes. In addition, these severe chronic wounds can lead to major disabilities and become a heavy financial burden owing to the lack of efficient therapeutic options.

Recent findings demonstrate that stem cells have therapeutic capacity in healing chronic wounds [2]. Mesenchymal stem cells (MSCs), endothelial progenitor cells, and hematopoietic stem cells are involved in the wound healing process via direct or indirect mechanisms, such as transdifferentiation, re-epithelialization, paracrine actions, and remodeling of the extracellular matrix [3,4,5]. However, there are some controversies regarding the efficacy of stem cells in clinical trials [6]. Thus, interdisciplinary combination approaches such as exosomes, biomaterials, and biological engineering are needed to develop drug delivery systems or stem cell therapies to treat chronic wounds [7,8].

Angiogenesis plays an important role in wound healing [9]. Stromal-cell-derived factor (SDF)1 and its receptor C-X-C chemokine receptor (CXCR)4 are associated with neovascularization through homing and migration of multiple stem cells [10,11]. In fact, CXCR4-overexpressing MSCs exhibit an accelerated wound healing capability by migrating to the injured skin area. A strong inflammatory reaction is present in the ulcer microenvironment, which leads to increased protease secretion and degradation or loss of stem cell activity. A potent anti-inflammatory cytokine, interleukin-10 (IL-10), plays an important role in the ability to recapitulate scarless healing in postnatal tissues [12].

To promote the healing of chronic wounds, we hypothesized that pro-angiogenic chemokine initiate angiogenesis and that anti-inflammatory factors can support the formation of a stable vasculature. In this study, we generated CXCR4/IL-10-expressing amniotic MSCs (AMM/CI) and aimed to investigate the therapeutic potency of dual angiogenic/anti-inflammatory gene knock-in stem cells in a chronic wound healing model.

## 2. Results

### 2.1. Generation of an CXCR4/IL-10 Knock-in AMM Line

**Figure 1:**
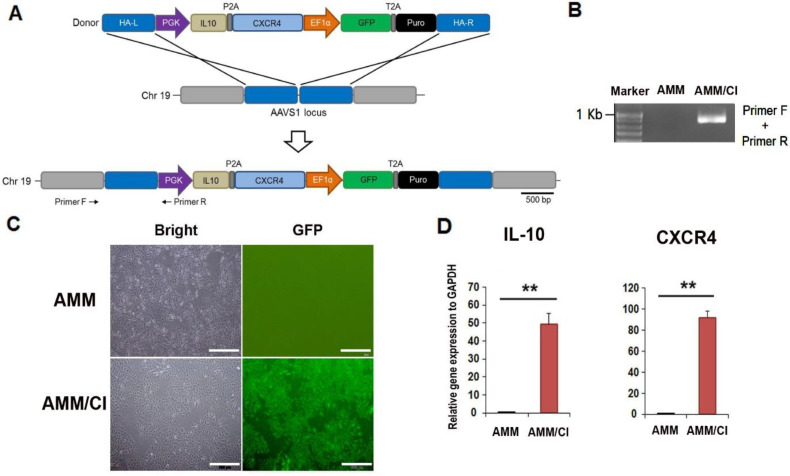
Generation and characteristics of the AMM/CI. (**A**) Schematic picture of the donor plasmid and vector carrying CXCR4 and IL10 targeting the AAVS1 locus. Primers F and R indicate primer lo-cations for junction detection. Abbreviations: HA-L, left homology arm; HA-R, right homology arm; PGK, phosphoglycerate kinase promoter; EF1α, elongation factor-1 alpha promoter; Puro, puromycin. (**B**) Result of junction PCR to confirm the correct knock-in of donor plasmid. (**C**) GFP expression in AMM/CI. Transfected AMM were incubated with puromycin for selection, then isolated by fluorescence-activated cell sorting (FACS) selection. Bars = 500 μm. (**D**) The confir-mation of CXCR4 and IL10 expression levels in the AMM/CI by qRT-PCR. ** *p* < 0.01, n = 4 in each group.

To generate a dual gene-expressing stem cell line using gene editing (TALEN), a safe locus on chromosome 19 (AAVS1) was chosen as the target site. The donor plasmid carried the PGK promoter, *CXCR4/IL-10*, and EF1α promoters, as well as GFP-T2A-puromycin (Figure 1A). Genomic DNA PCR was performed to evaluate the integration of the donor plasmid into the AMM genome. We then determined the correct insertion of the donor plasmid (Figure 1B). The donor vector was transfected into AMM cells, and approximately 99% of GFP-positive AMM/CI were selected via puromycin treatment and fluorescence-activated cell sorting (Figure 1C). Next, we confirmed IL-10 and CXCR4 expression in AMM/CI using qRT-PCR. Transfected AMM/CI expressed high levels of IL-10 and CXCR4 compared to the untreated AMM control, indicating the successful establishment of an AMM/CI cell line (Figure 1D).

### 2.2. Factors Secreted by AMM/CI Promoted Matrigel Tube Formation

**Figure 2:**
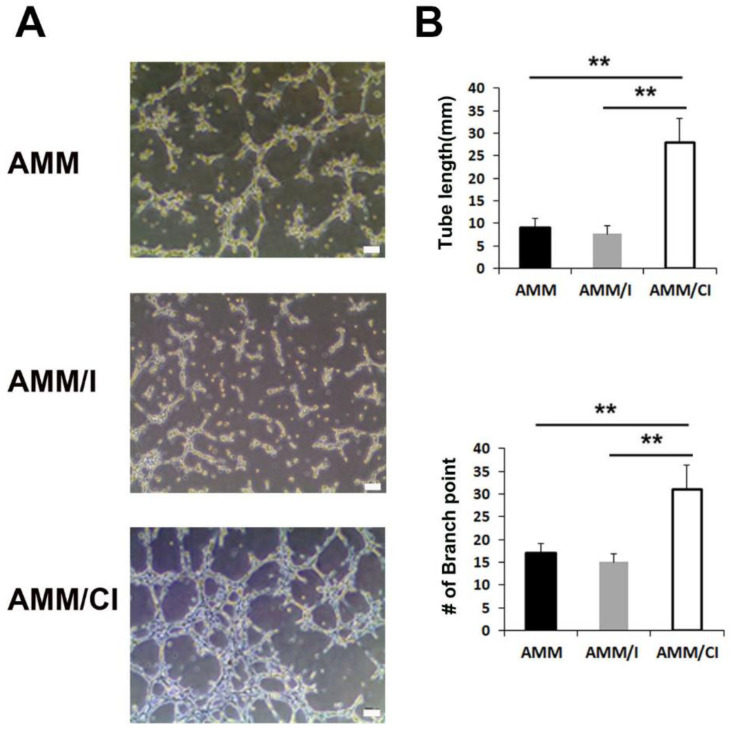
Matrigel tube formation of secreted factors derived from AMM/CI. (**A**) Representative pictures of Matrigel tube formation induced by CM of AMM, AMM/I, or AMM/CI for 3 h. Bars: 200 μm. (**B**) Quantification of Matrigel tube formation. Numbers of tube lengths and branching points were significantly higher in CM of AMM/CI compared with AMM or AMM/I. ** *p* < 0.01, n = 5 per group.

To evaluate whether protein factors secreted by AMM/CI enhance endothelial cell tube formation, we performed a Matrigel tube formation assay using culture medium (CM). The assay showed that AMM/CI CM significantly increased tube length and branching points compared to IL-10 overexpressing AMM (AMM/I) or AMM in the control group (Figure 2A,B). These data indicate that factors secreted by AMM/CI have a higher angiogenic capacity than those secreted by AMM/I or untreated control AMM cells.

### 2.3. Factors Secreted by AMM/CI Promoted Phagocytosis

**Figure 3:**
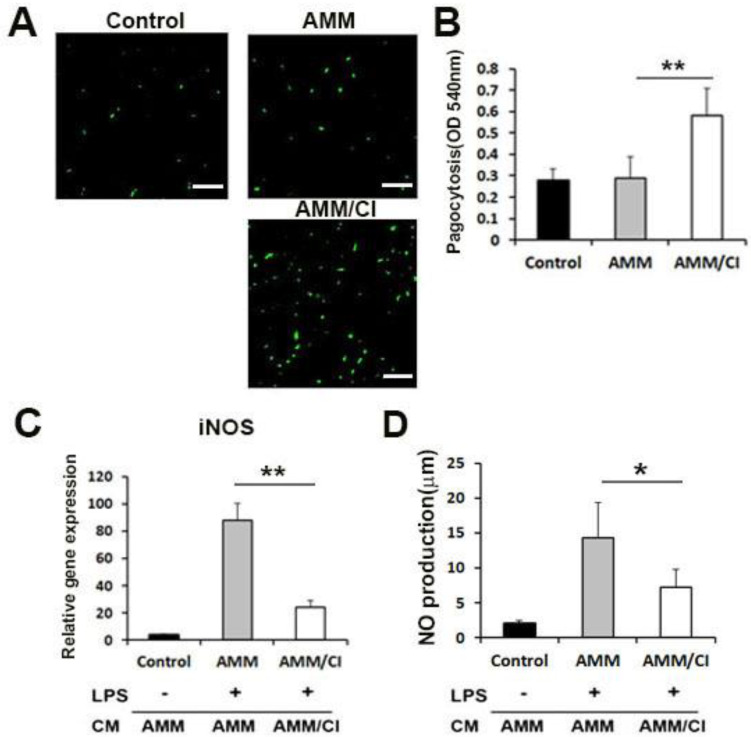
Phagocytosis and *in vitro* anti-inflammatory potential of secreted factors derived from AMM/CI. (**A**) Representative images of engulfing the fluorescent beads (green) in RAW 264.7 after treatment of vehicle control, CM of AMM, or AMM/CI. AMM/CI CM-treated cells exhibited high capacity in engulfing the fluorescent beads compared with the control or AMM CM group. Bars = 200 μm. (**B**) The neutral red experiment was performed to evaluate the effects of CM on the phagocytic potential of RAW 264.7. The neutral red experiment showed that the absorbance values of the RAW 264.7 were significantly higher in CM of AMM/CI than that of AMM or control group. ** *p* < 0.01, n = 5 per group. (**C**) The effect of AMM/CI CM on LPS-induced iNOS mRNA in RAW 264.7 cells. ** *p* < 0.01, n = 5 per group. (**D**) The effect of AMM/CI CM on LPS-induced NO production in RAW 264.7 cells. Cells were pre-treated with CM of each cell for 12 h and then incubated with LPS for 18 h. The amount of NO was measured by the Griess method. * *p* < 0.05, n = 5 per group.

The phagocytic potential of AMM/CI was evaluated using RAW 264.7 macrophage cells. RAW 264.7 cells treated with the CM of AMM/CI showed a higher capacity for engulfing fluorescent beads compared to control AMM CM (Figure 3A). In addition, the neutral red phagocytic assay showed that the absorbance values of the AMM/CI CM-treated group were significantly higher than the control AMM-CM-treated group (Figure 3B). These data indicate that the factors secreted by AMM/CI promote the phagocytic activity of macrophages.

### 2.4. Factors Secreted by AMM/CI Showed an Anti-Inflammatory Effect

To investigate the anti-inflammatory effect of AMM/CI, we examined the expression of the inflammatory factor iNOS in RAW 264.7 cells. The results revealed that AMM-CM- and LPS-treated macrophages exhibited increased levels of iNOS (Figure 3C). In contrast, macrophages treated with CM from AMM/CI and LPS exhibited lower iNOS expression compared to the control macrophages treated with AMM CM and LPS.

Next, we also investigate the LPS-induced NO production in RAW 264.7 cells. Interestingly, CM from AMM/CI significantly inhibited LPS-induced NO production in RAW 264.7 cells compared to the control macrophages treated with AMM CM and LPS (Figure 3D).

### 2.5. AMM/CI Strongly Promoted Wound Healing 

**Figure 4:**
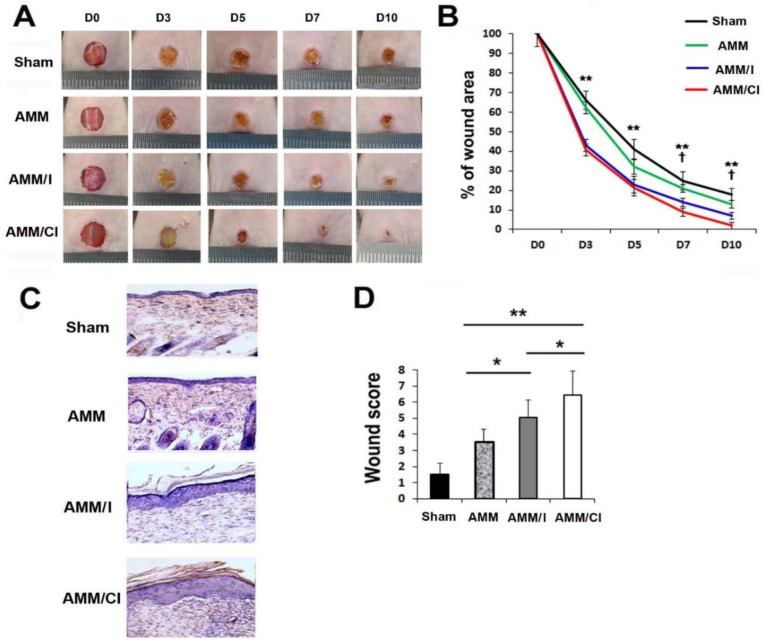
**AMM/CI transplantation showed robust therapeutic potential in the chronic wound healing mice model.** (**A**) Representative photographs of wound healing in excisional wound areas after cell injection. (**B**) Analysis of in vivo wound healing after cell injection. **^†^** *p* < 0.05, AMM/I vs. AMM/CI; ** *p* < 0.01, AMM vs. AMM/CI. n = 7 per group. (**C**) Representative wound histological pictures by H&E staining using cross-sectioned wound tissues at 10 days after cell injection. (**D**) Analysis of wound histological scores. ** *p* < 0.01, * *p* < 0.05, n = 7 per group.

To investigate the in vivo wound-healing capacity of AMM/CI, excisional wounding was performed in diabetes-induced nude mice. AMM, AMM/I, and AMM/CI were injected intradermally in five areas at a distance of 0.3 cm from the excisional wounds. Wound healing results revealed that AMM/CI-injected wounds showed accelerated wound recovery at days 7 and 10 compared to those injected with AMM or AMM/I (Figure 4A,B).

Next, histological scoring analysis was conducted by the criteria shown in Table 1. We used the amount of cytokeratin in the histological scoring analysis. Because cytokeratin is the intermediate filament protein expressed by epithelial cells, and epithelial cell proliferation and migration are fundamental processes to wound re-epithelialization. Histological analysis of wounds revealed that the wounds treated with AMM/CI exhibited enhanced re-epithelialization when compared to AMM or AMM/I cells (Figure 4C,D).

### 2.6. AMM/CI Induced Multiple Biological Factors for Wound Healing

**Figure 5:**
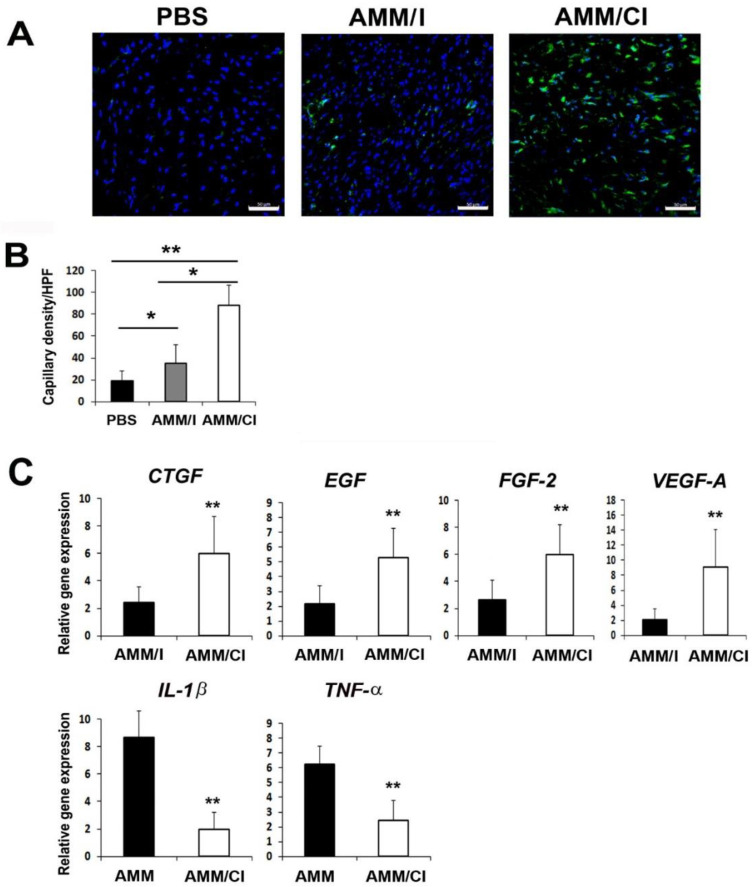
**AMM/CI transplantation enhanced the capillary density and expression of angiogenic factors in wound areas.** (**A**) Representative photographs of ILB4 (green), which is an endothelial cell marker, in the skin wounds at 10 days after cell injection. Nuclear (DAPI) staining is blue. Bars = 50 μm. (**B**) Quantification of ILB4-positive cells in the skin wounds at 10 days after cell transplantation. * *p* < 0.05, ** *p* < 0.01, n = 7 per group. (**C**) Analysis of levels of angiogenic and inflammation gene expression in wound tissues 5 days after cell injection. Various angiogenic and inflammation gene expression levels were measured by qRT-PCR. ** *p* < 0.01, n = 7 per group.

To elucidate the mechanisms relating to the therapeutic effects in wounds, we performed immunohistochemistry on wound tissue sections using endothelial cell markers. Capillary density in the wound tissues was also measured using the endothelial marker ILB4. Intriguingly, the AMM/CI-injected wound group showed a significantly higher capillary density than those injected with AMM or AMM/I (Figure 5A,B). Next, to further elucidate the mechanisms of wound healing, we examined the changes in the levels of angiogenic and inflammatory factors 5 days after cell injection in the wound tissues. qRT-PCR results revealed that AMM/CI injection significantly upregulated connective tissue growth factor (CTGF), EGF, fibroblast growth factor (FGF)-2, and vascular endothelial growth factor (VEGF)-A in wound tissues compared to those injected with AMM/I. Moreover, AMM/CI injection significantly downregulated interleukin (IL)-1β and tumor necrosis factor (TNF)-α, indicating that transplantation of AMM/CI induces multiple biological factors in the wound healing process (Figure 5C).

### 2.7. AMM/CI Maintained High Re-Epithelialization Potential in Wounds

**Figure 6:**
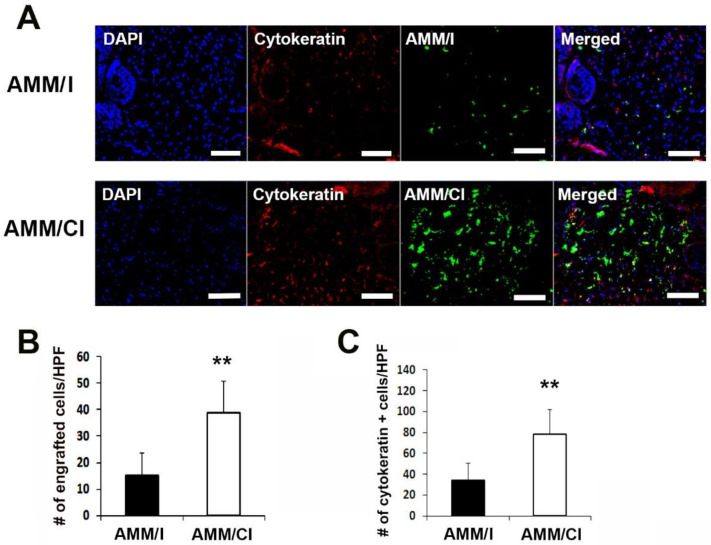
**AMM/CI showed high engraftment and epithelization potentials in wound tissue.** (**A**) Representative images of cytokeratin immunostained wound tissue sections 10 days after cell injection. Bars = 200 μm. (**B**) Quantification of engrafted cells in the wound area after cell injection. ** *p* < 0.01, n = 7 per group. Engrafted AMM/CI were measured in the skin wound at 10 days after cell transplantation. (**C**) Quantification of cytokeratin-expressing cells in skin wound tissues. Cytokeratin-positive cells were measured in the skin wound tissues at 10 days after cell transplantation. ** *p* < 0.01, n = 6 per group.

The engraftment potential of AMM/CI was measured in the wound. Immunohistochemical results showed that AMM/CI showed a significantly higher engraftment potential in wound areas than AMM/I (Figure 6A,B).

In addition, immunostaining for epithelialization revealed that the level of cytokeratin immunoreactivity was significantly higher in the AMM/CI-injected wound group than that in the AMM/I-injected group I (Figure 6A,C).

## 3. Discussion

In the present study, we report the characteristics and therapeutic potential in wound healing of dual angiogenic and anti-inflammatory-gene-expressing AMM/CI. The major findings are the following: (1) secreted factors from AMM/CI showed angiogenic and anti-inflammatory properties in vitro, and (2) the injection of AMM/CI in vivo accelerated wound healing through re-epithelialization and angiogenesis. Collectively, these data suggest that dual gene editing of CXCR4/IL-10 in AMM cells could be a novel strategy to induce robust skin regeneration.

Many chronic diseases related to obesity or diabetes can cause refractory wounds owing to continuous inflammation, hypoxia, infection, and vascular insufficiency [13]. Repair of chronic wounds by conventional therapies remains challenging [14]. There have been novel efforts to repair non-healing or chronic wounds using stem cells [15]. However, the therapeutic efficacy of stem cells remains controversial and their efficacy in enhancing tissue repair needs to be improved. Recent advances in genome editing and stem cells technology have prompted investigators to envisage the generation of safe and efficient advanced therapy for the treatment of incurable diseases [16]. Thus, we also attempted to identify a novel, safe, and more effective method using gene editing and stem cells to induce tissue regeneration.

The SDF-1 receptor CXCR4 is a potent angiogenic factor. The CXCR4/CXCL12 (SDF-1) axis enhances metastasis by mediating the migration and proliferation of tumor cells and inducing angiogenesis via the Akt signaling pathway. CXCR4 overexpression in MSCs resulted in increased angiogenesis and neuroprotection in a stroke animal model. Overexpression of CXCR4 improved stem cell engraftment, survival, and tissue regeneration in mouse hindlimb ischemia, and CXCR4-overexpressing bone marrow MSCs accelerate wound healing by migrating to the injured skin. In line with this report, we found that CXCR4-expressing AMM cells showed high cell engraftment in the wound area and accelerated wound healing.

IL-10 is the representative anti-inflammatory factor that regulates neutrophilic infiltration and various pro-inflammatory mediators. IL-10 is an essential factor for scarless regenerative wound healing because of its low inflammatory function [17]. IL-10-gene-modified AMM cells show increased regenerative wound healing through multiple biological effects, such as enhancing angiogenesis, modulating inflammation, and regulating extracellular matrix remodeling [18]. In addition, IL-10-modified adipose MSCs prevent hypertrophic scar formation by regulating inflammation [19]. In line with these reports, our results showed that secretory factors derived from AMM/CI regulated phagocytosis and anti-inflammation in macrophages, indicating favorable effects on wound healing via the regulation of the inflammatory response.

Macrophage dysfunction impairs the resolution of inflammation in diabetic mice wounds [20]. To prevent inflammation, macrophages remove neutrophils by phagocytosis during the inflammation stage, followed by the induction of neutrophil apoptosis [21]. Therefore, we examined the effects of AMM/CI on macrophages. Interestingly, the factors secreted by these cells activated phagocytosis and inhibited iNOS gene expression in LPS-induced macrophages, suggesting that AMM/CI are associated with anti-inflammation during the inflammatory stage of wound injury. However, more studies are necessary regarding the response of cellular infiltration of inflammatory cells.

When the skin tissue is damaged, the inflammatory response is initiated by neutrophil macrophage effusion and aggregation, induced by many pro-inflammatory chemokines or cytokines such as SDF-1 [22], followed by the secretion of large amounts of tissue-regenerating factors [23]. Our results imply that macrophages induced by IL-10 deriving from AMM/CI might reduce the inflammatory response. In addition, increased levels of SDF-1 in the wound tissue may enhance the survival or engraftment potential of AMM/CI, promoting synergistic therapeutic effects in wound healing.

Multiple angiogenic factors or cytokines secreted by transplanted stem cells play a crucial paracrine role in the regeneration of host tissues [24]. It has been previously demonstrated that the major therapeutic mechanism behind stem cell injection is an increase in pro-angiogenic or anti-inflammatory factors [25]. In this study, the injection of AMM/CI enhanced the levels of angiogenic and anti-inflammatory factors in the wound tissues. Thus, in agreement with previous reports, the overexpression of the dual factors IL-10 and CXCR4 might synergistically stimulate regeneration in damaged skin tissue.

In conclusion, this study demonstrated that overexpressing angiogenic chemokine CXCR4 promotes angiogenesis and anti-inflammatory factor IL-10, supporting the formation of a stable vasculature. Thus, the strategy of dual or multiple angiogenic/anti-inflammatory factor overexpression in stem cells could be a novel technology to promote synergistic therapeutic effects for tissue repair. In specific, treatment with AMM/CI could be an alternative approach to obtain enhanced therapeutic effects in chronic wound disease. However, further studies are necessary to evaluate their efficacy in clinical applications.

## 4. Materials and Methods

### 4.1. Cell Culture

Human umbilical vein endothelial cells (HUVECs) and RAW 264.7 cells were obtained from ATCC (Manassas, VA, USA). Human amniotic mesenchymal stem cells (AMM) were obtained from Thermo Scientific, Inc. (Watlham, MA, USA). AMM were grown in low-glucose Dulbecco’s modified Eagle’s medium (LG-DMEM; Gibco, Grand Island, NY, USA) with 10% fetal bovine serum (FBS), 100 U/mL penicillin, and 100 μg/mL streptomycin (Gibco), as described previously. HUVECs were grown in endothelial growth medium (EGM-2) complete medium (Lonza, Walkersville, MD, USA).

### 4.2. Donor Construction, Transfection and Selection

IL10-T2A-CXCR4 or IL10 was synthesized and inserted into the AAVS1 safe-harbor-site-targeting donor vector (System Biosciences, Palo Alto, CA, USA) at the NdeI and SalI restriction sites according to a previous report [26]. For electroporation, 2 × 10^5^ AMM were resuspended with AAVS1 left TALE-Nuclease vector (System Biosciences), AAVS1 right TALE-Nuclease vector (System Biosciences), and AAVS1 HR Donor vector (System Biosciences), and they were electroporated by a Neon Transfection System (Thermo Fisher Scientific, Waltham, MA, USA). Six days after transfection, IL10/CXCR4 or IL10 knock-in AMM (AMM/I) were selected by incubating with 6 μg/mL puromycin for 12~15 days [27]. After puromycin selection, green fluorescent protein (GFP)-expressing AMM were sorted on S3e Cell Sorter (Bio-Rad, Hercules, CA, USA). Next, genomic DNA from transfected AMM was isolated using a Total DNA Extraction Mini Kit (Intron Biotechnology, Seoul, Korea) and touch-down PCR (36 cycles) was used for the genomic DNA amplification as previously described [26].

### 4.3. Quantitative Real-Time (qRT)-PCR Analysis

qRT-PCR was performed according to a previous report [28]. In brief, the total RNA was extracted from cells using RNA-Stat reagent (Iso-Tex Diagnostics, Friendswood, TX, USA), and the concentrations of RNA were determined by a NanoDrop ND-1000 spectrophotometer (NanoDrop Technologies, Wilmington, DE, USA). Isolated RNA was reverse-transcribed using TaqMan Reverse Transcription Reagents (Applied Biosystems, Foster City, CA, USA). The synthesized cDNA was subjected to qRT-PCR using primers and probes. The relative mRNA expression was normalized to GAPDH expression. The qRT-PCR primers and probe sets were purchased from Applied Biosystems, and the genes and catalog numbers were as follows: for human IL-10 (Hs00237017_m1), CXCR4 (Hs00976734_m1) and GAPDH (Hs99999905_m1); for mouse iNOS, CAT GCT ACT GGA GGT GGG TG (forward), CAT TGA TCT CCG TGA CAG CC (reverse), IL-1β (Mm00434228_m1), tumor necrosis factor-α (TNF-α) (Mm00443259_g1), fibroblast growth factor-2 (FGF-2) (Mm01285715_m1), vascular endothelial growth factor A (VEGF-A) (Mm01281448_g1), and GAPDH (Mm99999915_g1). All of the primer sets were obtained from Applied Biosystems.

### 4.4. Culture Medium Preparation

Culture media (CM) was prepared as described previously [29]. Briefly, 2 × 10^6^ cells were seeded into T-75 cell culture flasks and grown for 48 h until the cells reached approximately 95% confluence. The CMs were centrifuged, and the supernatants were harvested and used for this study.

### 4.5. Matrigel Tube Formation Assay

HUVECs at a concentration of 1 × 10^4^ cells/well were embedded in each CM derived from each cells. Representative fields were photographed using microscopy after 12 h of incubation. Tube length and number of branching points were examined as previously reported [30].

### 4.6. Phagocytic Assay

RAW 264.7 was cultured in a 24-well plate and treated with CM of AMM or AMM/CI for 24 h. Then, Latex Beads-Rabbit IgG-FITC solution (Cayman Chemical, Ann Arbor, MI, USA) was loaded and cultured for 8 h, and CM was changed to observe the fluorescently labeled latex beads using a florescent microscope. RAW 264.7 cultured without CM was used as the control group.

### 4.7. Neutral Red Phagocytic Activity

RAW 264.7 cells were treated with CMs of each cell or a 0.5 μg/mL LPS in a 96-well plate for 24 h and loaded with 100 μL of 0.075% neutral red solution (Sigma, St. Louis, MO, USA). Each AMM or AMM/CI CM was treated in RAW 264.7 cells for 24 h, and then the RAW 264.7 cells were cultured at 37 °C for 1 h. Following this, the cells were washed, and the absorbance value at 540 nm was measured using an enzyme immunoassay analyzer (Epoch, BioTek Instruments, Winooski, VT, USA).

### 4.8. Nitric Oxide (NO) Assay

NO assay was performed as previously described [31]. In brief, 1 × 10^6^ RAW 264.7 cells were cultured in 6-well plates using CMs for 12 h, followed by LPS (1 μg/mL) for 15 h. The cell-free supernatant was harvested and mixed with Griess reagent for 10 min. The absorbance of the mixture was examined at 540 nm, and NO level was measured by using sodium nitrite as the standard.

### 4.9. Generation of Type 1 Diabetes Mice Model

All experimental protocols were approved by the Catholic Kwandong University Institutional Animal Care and Use Committee, and all procedures were conducted in accordance with the Guide for the Care and Use of Laboratory Animals published by the U.S. National Institutes of Health (NIH Publication No. 85-23). Male nude mice (Joongang Laboratory Animal Inc., Seoul, Republic of Korea) at 12~14 weeks old were intra-peritoneally injected with 45 mg/kg streptozotocin (STZ; Sigma, St. Louis, MO, USA) dissolved in 50 mM sterile citrate buffer (0.05 M sodium citrate, pH 4.5). STZ or citrate buffer (sham) was injected for 5 consecutive days during the first week. Four weeks after injection, blood glucose level was measured. Mice with glucose levels > 280 mg/dL were used for this study.

### 4.10. Excisional Wound Mouse Model and Cell Injection

All animal protocols were approved by the Institutional Animal Care and Use Committees of the Laboratory Animal at the Catholic Kwandong University, and all procedures were performed in accordance with the Guide for the Care and Use of Laboratory Animals. Male nude mice were randomly divided into four groups: sham (control, n = 6), AMM-injected (AMM, n = 6), AMM/I-injected (AMM/I, n = 6), and AMM/CI-injected (AMM/CI, n = 6). An excisional wound mouse model was induced as previously described [29]. In brief, mice were anesthetized by intraperitoneal injection of 3.5% chloral hydrate (1 mL/100 g), and 6 mm full-thickness excision skin wounds were generated using a surgical punch. Each group of cells (1 × 10^6^ cells suspended in 100 µL PBS) was injected intradermally in five areas around the wound. The efficiency of wound closure was evaluated on 0, 5, 7, and 10 days after cell injection.

### 4.11. Wound Analysis

Wound analysis was conducted as previously described [29]. The wound area was measured in a blinded manner using pictures. The ratio of wound area was calculated as (original wound area—new wound area)/original wound area × 100.

### 4.12. Histological Analysis

Histological analysis was performed as previously described [29]. Mice were euthanized at 14 days, and skin wound samples were collected. The tissue specimens were fixed with 4% paraformaldehyde for one day and embedded with OCT compound (Sakura Finetek USA, Torrance, CA, USA). Sections (10 μm) were stained with hematoxylin and eosin (H&E). For immunofluorescence, tissue sections were stained with primary mouse anti-cytokeratin (1:300; Abcam, Cambridge, MA, USA) and Cy3-conjugated secondary antibody (1:300, Jackson ImmunoResearch, West Grove, PA, USA). Frozen sections were also stained with primary biotinylated ILB4 (1:200; Vector Laboratory Inc., Burlingame, CA, USA) and secondary strepta-avidin Alexafluor 488 (1:300; Invitrogen). Nuclei were stained with 4′,6-diamidino-2-phenylindole (DAPI; Sigma), and six fields from each tissue section were randomly selected and counted.

### 4.13. Statistical Analysis

All data are presented as the mean ± standard deviation. Statistical analyses were performed using the Student’s *t*-test and ANOVA with Bonferroni’s multiple comparison test using SPSS v12.0. *p* < 0.05 was considered statistically significant.

## Figures and Tables

**Table 1:** Criteria for histological scores.

Score	Dermal and Epidermal Regeneration	Granulation Tissue Thickness	Re-Epithelization (10 Days Wounds Only)
**1–3**	Little epidermal and dermal organization	Thin granulation layer	Cytokeratin+ cells < 100/mm^2^
**4–6**	Moderate dermal and epidermal organization	Moderate granulation layer	Cytokeratin+ cells 100~200/mm^2^
**7–9**	Complete remodeling of dermis and epidermis	Thick granulation layer	Cytokeratin+ cells < 300/mm^2^

## Data Availability

Not applicable.
